# Development of the Digital Dementia Scale Adult Form (DDAF) and examination of its psychometric characteristics

**DOI:** 10.1007/s11845-026-04446-4

**Published:** 2026-05-19

**Authors:** Yalcin Kanbay, Aydan Akkurt Yalcinturk, Elcin Babaoglu Gulseven, Aysun Akcam

**Affiliations:** 1 University of Health Sciences, Hamidiye Faculty of Nursing, Department of Psychiatric Nursing, Istanbul, Turkey; 2 Artvin Coruh University, Health Sciences Faculty, Department of Psychiatric Nursing, Artvin, Turkey; 3 Uskudar University, Faculty of Health Sciences, Department of Nursing, Istanbul, Turkey; 4 Necmettin Erbakan University, Faculty of Education, Department of Turkish Language Teaching, Konya, Turkey

**Keywords:** Digital dementia, Excessive screen time, Reliability, Validity

## Abstract

**Aim:**

Developing a measurement tool to determine digital dementia is an important step toward understanding the prevalence and effects of digital dementia. The present study aimed to develop the “Digital Dementia Scale Adult Form” to determine digital dementia in individuals aged 18 and over by examining the concept of digital dementia.

**Methods:**

The form which consisted of 22 statements with proven validity in terms of language and psychometrics was applied to 615 individuals. Exploratory and confirmatory factor analysis was used to determine the construct validity, and a 27% lower-upper group comparison was made to determine the internal validity

**Results:**

The “Digital Dementia Scale Adult Form” consisted of 3 subdimensions and 15 items and could explain 65.7% of the total variance as a 5-point Likert-type scale (Never: “0”, Rarely: “1”, Sometimes: “2”, Frequently: “3”, and Always: “4”). The total score on the scale varies between 0-60, and as the score increases, the level of digital dementia also increases. The Cronbach α Reliability Coefficient of the scale was calculated as .92, indicating high reliability.

**Conclusion:**

The “Digital Dementia Scale Adult Form” is valid in terms of scope and content and has high reliability.

## Introduction

Dementia is the general name for brain syndromes that have negative effects on memory, thinking, behaviors, and emotions as a rapidly increasing public healthcare concern on a global scale [1]. According to the World Health Organization 2021 data, approximately 50 million people live with dementia on a global scale, and nearly 10 million new cases are diagnosed each year [2]. It is expected that this number will exceed 150 million by 2050 with the aging population [3]. The prevalence of dementia is also increasing rapidly in Turkiye, especially with the increase in the elderly population. There are approximately 1 million patients with dementia in Turkiye and this number is expected to reach 3 million by 2050 [4]. Estimates of the increase in dementia rates in the future are based on factors such as the increasing elderly population and life expectancy [5]. Considering the predictions that dementia rates will increase in the future, it must be taken into account that digital dementia may also contribute to this increase [6, 7]. The concept of digital dementia was introduced by German neurologist Manfred Spitzer. Since the release of Dr. Spitzer’s *Digital Dementia*, a book focusing on the effects of gaming on children, the phrase has become more widely used. The concept of digital dementia describes a condition in which excessive use of digital devices leads to impaired cognitive functioning. Spitzer argued that the use of digital media can have long-term detrimental effects. These effects take the form that signs of neurodegeneration (the progressive loss of neurons, either structurally or functionally), which should be expected in old age, are increasingly seen in adults. Digital dementia differs from organic dementia (e.g., Alzheimer’s) in that organic dementia results from structural brain pathology, while digital dementia is conceptualized as functional cognitive fatigue and cognitive externalization associated with excessive screen use [8]. Another difference between them relates to the prognosis of the disorder. Organic dementias are irreversible brain disorders that progress from early to late stages. In digital dementia, cognitive function can be improved by providing the brain with a digital detox (avoiding excessive device use) and providing a chance to rest [9, 10].

Evidence obtained from biopsychosocial studies suggests that chronic sensory stimulation through excessive screen exposure impacts brain development and increases the risk of cognitive, emotional., and behavioral disorders in adolescents and young adults suggests that some of these effects are similar to those seen in adults with mild cognitive impairment in the early stages of dementia, which include distractibility, disorientation, recent memory acquisition (anterograde amnesia), recall of memories (retrograde amnesia), social functioning, and self-care. Excessive screen time is also known to alter gray matter and white matter volumes in the brain, increase the risk of mood disorders, and impair memory acquisition and learning, which are known risk factors for dementia. Chronic sensory overstimulation during brain development (i.e., excessive screen time) increases the risk of accelerated neurodegeneration (i.e., amnesia, early-onset dementia) in adulthood [11].

The number of studies conducted on digital dementia is increasing in the world. Global studies conducted in this field report important findings about the prevalence and effects of digital dementia. For example, a study conducted in the United Arab Emirates reported that participants who had higher nighttime screen exposure had lower cognitive scores in the areas of information processing speed, working memory, calculation, and attention. The study also emphasized the importance of determining future recommended screen time guidelines for young adults and stated that healthy cognitive habits must be encouraged to preserve cognitive functions and reduce the risk of developing neurodegenerative diseases in the future [12]. Another study that examined the effects of prolonged screen time in 14 countries and 44 articles generally associated increased screen time with negative outcomes such as lower self-esteem, increased mental healthcare concerns, incidence, and severity of addictions, slowed learning and acquisition, and risk of early cognitive decline [13].

There are studies examining the negative impacts of behaviors that increase the use and duration of digital technologies such as digital media, social media, messaging applications, multitasking habits, and e-mail checking frequencies. Rosen, Carrier and Cheever (2013) reported that social media and messaging applications could cause distraction and difficulty switching tasks [14]. Ophir, Nass and Wagner (2009) reported that media multitasking users’ cognitive control abilities weakened [15]. Kushlev and Dunn (2015) found that increasing the frequency of e-mail checking increased stress levels [16]. Wilmer, Sherman, and Chein (2017) reported that smartphone use could potentially have a deteriorating effect on cognitive functions [10].

Although there are studies conducted on digital dementia at the global level, the subject of digital dementia has not yet been sufficiently researched in Turkiye, and more studies are needed in this field. The intensive use of digital devices in our country and the fact that a large part of the young generation, especially, is immersed in digital media increases the risk of digital dementia. According to the 2022 Turkish Statistical Institute Household Information Technology Usage Survey data, the rate of households with internet access in Turkiye was 94.1% and the rate of regular internet users was 85% [17]. For this reason, comprehensive studies are needed to determine the prevalence and effects of digital dementia in Turkiye.

The literature suggests that excessive use of digital devices is associated with distraction, memory problems, cognitive decline, and emotional disorders. The negative effects of digital behaviors (especially social media, messaging applications, and multitasking habits) on cognitive functions have been emphasized. Also, biopsychosocial evidence on how excessive screen exposure affects brain development and cognitive functions suggests that digital dementia might be a significant healthcare concern in young adults as well as in the aging population. Considering that studies on digital dementia are limited in Turkiye, developing a measurement tool to determine digital dementia is an important step toward understanding the prevalence and effects of digital dementia. The present study aimed to contribute to the literature in this field and support the development of digital healthcare policies by determining the psychometric characteristics of the Digital Dementia Scale Adult Form (DDAF).

## Materials and methods

### Purpose and type of study

The study had a methodological design and aimed to develop the “Digital Dementia Scale Adult Form” that would enable data collection based on individuals’ perceptions in both clinical and field studies.

### Development of the Digital Dementia Scale Adult Form (DDAF)

#### Determination of the appropriate scale to meet the need

When the literature was reviewed, concepts that could be associated with digital dementia were detected, but it was found that there was no measurement tool to measure the digital dementia levels of individuals [8, 11, 16]. To meet this need in the literature, it was planned to develop a Digital Dementia Scale Adult Form in this study.

#### Creation of the question pool

The question pool of the Digital Dementia Scale Adult Form was created based on a comprehensive literature review and expert opinions. In the first stage, existing studies and theoretical models in the literature were reviewed to determine the effects of digital dementia on cognitive functions, memory, attention, and daily living activities [18–20]. In the second step, the scope and contents of the scale were confirmed and expanded by obtaining opinions from psychologists, neurologists, and education experts who were experts in digital dementia. The basic components and symptoms of digital dementia were determined based on the literature review and expert opinions and the conceptual framework of the scale was formed. In the evaluation made in this way, it was found that the concept of digital dementia consisted of elements such as memory and cognitive abilities, attention and concentration, learning and information processing, social skills, physical and emotional health, lifestyle, and habits. An item pool was created in line with these sub-concepts and 42 statements were collected. These items were evaluated by the researchers and reduced to 22 items.

#### Content validity

For the content validity of the statements that could be included in the scale planned to be developed, Content Validity Rate (CVR) and Content Validity Index (CVI) were calculated by consulting experts. For content validity, expert opinions were obtained from a team of 9 experts in the fields of nursing, measurement and evaluation, psychology, and Turkish language.

#### Pilot implementation

To determine whether the trial form, which had been validated for content, was understood correctly by the sample, the form was applied to a sample of 50 people. During the pilot implementation, the understandability of the questions, language and expression problems, response times, and general consistency were evaluated and the questions were revised in line with the feedback obtained. Pilot implementation data were subjected to statistical analysis, and the questions’ discrimination power, internal consistency, and contribution to the total scale score were examined.

#### Population and sample

Firstly, Exploratory Factor Analysis (EFA) was made to uncover the structure and then Confirmatory Factor Analysis (CFA) was used to verify the emerged structure. For this reason, two separate samples with similar characteristics were needed in the study. As a result of the data collection, a sample of 615 people (312 for AFA and 303 for CFA) aged 18 and over who use digital devices such as smartphones, tablets, computers, televisions, etc. was reached. A total of 55.1% of the AFA sample was female, 67.3% were married, and the mean age of the sample was 41.9 ± 14.1. The average daily duration of digital device use is 5.1 ± 3.1 h. Also, 55.4% of the CFA sample was female, 67.7% were married, and the mean age of the sample was 42.2 ± 12.1. The average daily duration of digital device use is 4.9 ± 2.6 h.

Inclusion Criteria

- Using digital devices such as smartphones, tablets, computers, televisions, etc.
- Being 18 years old or older.
- Agreeing to participate in the study.

Exclusion Criteria

- Not using digital devices such as smartphones, tablets, computers, televisions, etc.
- Being under 18 years of age.
- Refusing to participate in the study.

### Data collection tools

The “Descriptive Data Form” and “Digital Dementia Scale Adult Form-Draft” were used to determine the descriptive characteristics of the participants in the study.

#### Descriptive data form

There were 3 questions on the age, gender, and marital status of the participants.

#### Digital dementia scale adult form-draft

There were 22 items in the draft form and were rated as 0 = Never, 1 = Rarely, 2 = Sometimes, 3 = Often, and 4 = Always. The questionnaire form includes an instruction section to help participants answer the questions correctly. In this section, digital tools are explained. In addition, the following statement is provided: “Below are various situations that may arise due to spending time in front of a screen or using digital devices. Please indicate how often you experience these situations in a way that best reflects you.”

### Data collection

In the data collection, Google Forms and face-to-face interview methods were used. Google Forms is an online tool that facilitates reaching large audiences and allows participants to fill out the survey in their own time. In this way, participants from 7 geographical regions of Turkiye were reached.

### Evaluation of data

The SPSS 26 package program was used for descriptive statistics and EFA, and the AMOS 23 package program was used for CFA in the data analysis. Number, mean, and percentage values were given for descriptive statistics. The correlation analysis was used to search for relationships, and the construct validity of the scale was carried out with Exploratory Factor Analysis (EFA) and Confirmatory Factor Analysis (CFA). The Average Variance Extracted (AVE) values of the items should be 0.50 or higher, and the Composite Reliability (CR), also referred to as construct reliability, should be 0.70 or higher, which are considered acceptable thresholds. Cronbach alpha coefficient and split-half test reliability were calculated for the reliability of the scale.

## Results

In the present study, the Lawshe Technique was used for the content validity of the measurement tool planned to be developed. The technique uses expert opinions to evaluate how appropriate the items of a measurement tool are. The opinions of 9 experts were obtained for the content validity of the scale. The critical value calculated for 9 experts was 0.778. When the items were examined, it was found that the CVR values of items I20, I21, and I22 were lower than the critical value. For this reason, these items were removed from the study (e.g., M_22_ “Checking incoming messages, warnings or notifications”. The content validity index was calculated on the remaining 19 items. According to the calculations, the Content Validity Index of the form was 0.953 (Table 1).

**Table 1:** Content validity findings

Number	Nu	N	CVR	Critical Value	Result		Number	Nu	N	CVR	Critical Value	Result
**I1**	9	9	1	0.778	+		**I12**	9	9	1	0.778	+
**I2**	9	9	1	0.778	+		**I13**	9	9	1	0.778	+
**I3**	8	9	0.78	0.778	+		**I14**	9	9	1	0.778	+
**I4**	8	9	0.78	0.778	+		**I15**	9	9	1	0.778	+
**I5**	9	9	1	0.778	+		**I16**	9	9	1	0.778	+
**I6**	9	9	1	0.778	+		**I17**	9	9	1	0.778	+
**I7**	9	9	1	0.778	+		**I18**	9	9	1	0.778	+
**I8**	9	9	1	0.778	+		**I19**	9	9	1	0.778	+
**I9**	8	9	0.78	0.778	+		*I20	7	9	0.56	0.778	**-**
**I10**	9	9	1	0.778	+		*I21	6	9	0.33	0.778	**-**
**I11**	8	9	0.78	0.778	+		*I22	7	9	0.56	0.778	**-**
Number of Experts = 9												
Critical Value=0.778												
CVI=0.953												

* Items that did not provide content validity and were removed from the study because experts gave the opinion “Not Appropriate” or “Needs Correction”, Nu: Number of experts who gave the opinion “Necessary” to the item, N: Number of experts who gave their opinion to the item, CVR (Nu-N/2)/(N/2): Content Validity Rate, CVI: Content Validity Index

When the item-total correlations of the items were examined, it was found that all items had values greater than the limit value of 0.30 (Table 2). For this reason, it was decided that all items analyzed must remain on the scale.

**Table 2:** Item-total correlations

Number	ITC	Number	ITC	Number	ITC	Number	ITC
**I1**	0.616	**I6**	0.591	**I11**	0.656	**I16**	0.699
**I2**	0.578	**I7**	0.627	**I12**	0.460	**I17**	0.712
**I3**	0.579	**I8**	0.563	**I13**	0.546	**I18**	0.632
**I4**	0.648	**I9**	0.706	**I14**	0.564	**I19**	0.671
**I5**	0.621	**I10**	0.708	**I15**	0.705	**….**	….

*ITC,* Item Total Correlation

The KMO value was determined to be 0.913. Bartlett’s Sphericity Test yielded a significant result (x^2^ =2712.796; *p*<.001). In light of these, it was decided that the data matrix was suitable for factor analysis (Table 3).

**Table 3:** EFA findings regarding the factor structure of the scale

Number	Item	F1	F2	F3
I1	Postponing daily tasks.	0.658		
I2	Staying in front of the screen for long periods.	0.799		
I3	Irregular, excessive, or inadequate eating and drinking.	0.638		
I4	Irregular, excessive, or inadequate sleep.	0.804		
I5	Spending time in front of the screen until late hours.	0.829		
I7	Waist, back, or neck pain.		0.744	
I8	Headaches or eyestrain.		0.853	
I9	Difficulty concentrating.		0.649	
I10	Mental fatigue.		0.737	
I11	Physical fatigue.		0.738	
I14	Decreased face-to-face social interaction.			0.650
I16	Feeling more restless or anxious.			0.699
I17	Feeling more depressed.			0.608
I18	Difficulty remembering everyday information or tasks.			0.852
I19	Difficulty learning or remembering new information.			0.782
	**Variance Explained**:	**48.11**	**9.56**	**7.32**
	**Total Variance**:	**65.7**		
	**KMO: 0.913; Bartlett’s: 2712**,**796; df: 105; p: 0.000**			

**F1**: Disruption in Daily Routine, **F2**: Physical and Mental Disruption, **F3**: Emotional and Cognitive Disruption

For the EFA that was conducted to determine the construct validity of the scale, the Principal Components Analysis was used, and as the factor rotation technique, the Direct Oblimin Technique from the Oblique Rotation Techniques was used based on the assumption that the factors were associated with each other, and rotation was performed in 9 iterations. To determine the factorization in the scale, factors with an eigenvalue greater than 1 were taken into consideration, and the line graph was examined. It was preferred that the variance rate explained by each factor formed be at least 5% and that the variance explained in the total of the scale be at least 50%. The item factor loadings being at least 0.50 was taken as a basis in the item elimination process from the factors, and in cases where the difference between the loadings of an item on two factors was less than 0.10, these items were considered overlapping items and removed from the study.

A 3-factor structure was obtained as a result of the analysis and some items (I_12_ and I_13)_ were evaluated as overlapping items (e.g., I_12_ “Using multiple digital devices at the same time”), and some items (I_6_ and I_15_) were removed from the scale because their factor loadings were below 0.50 (e.g., I_15_ “Feeling as if the mind is frozen”). As a result of item elimination, a 15-item structure was created. The total variance explained by the scale was calculated as 65.7%. The variance explained by Factor 1 was 48.1%, Factor 2 was 9.6%, and Factor 3 was 7.3%. (Table 3).

The internal validity of the items forming the structure of the scale was tested with a 27% lower-upper group comparison. The test scores obtained from the scale were ranked from smallest to largest, and it was found that 27% of the sample was 82 people. After this step, whether the difference between the lower group and the upper group was significant was evaluated with the T-Test in Independent Groups. A 27% lower group/upper group comparison was made for the internal validity of the 3-factor structure of the scale. The comparisons were made in the sub-dimensions of the scale and the total score and it was found that the mean scores of the 27% upper group with the highest mean score and the 27% sub-group with the lowest mean score showed statistically significant differences from each other both in the sub-groups and in the total scale (Table 4).

**Table 4:** Comparison of the 27% lower-upper group

Factor	Group	n	Mean	Standard Deviation	t	*p*
F1. Disruption in Daily Routine	Lower group	84	2.00	1.19	-32.287	0.000
	Upper Group		11.43	2.40		
F2. Physical and Mental Decline	Lower group	84	2.85	1.45	-37.669	0.000
	Upper Group		13.10	2.03		
F3. Emotional and Cognitive Decline	Lower group	84	1.25	1.03	-35.855	0.000
	Upper Group		11.37	2.37		
Digital Dementia	Lower group	84	7.90	3.30	-34.923	0.000
	Upper Group		33.81	5.94		

To test the reliability of the scale, Cronbach α Reliability Coefficient and Split-Half Test Consistency were calculated. Cronbach α reliability coefficient was calculated for the factors and the overall scale. The Disruption in Daily Routine factor was 0.85, the Physical and Mental Impairment factor was 0.87, and the Emotional and Cognitive Impairment factor was 0.87. The Cronbach α reliability coefficient calculated for the overall scale was 0.92 (Table 5). Also, the two-half-test consistency of the scale was calculated for the reliability of the scale. To apply this method, the items in the scale were listed, the odd-numbered items were grouped in one group and the even-numbered items were grouped in a separate group. Then, the total scores of these groups were obtained and the correlation between the two groups was examined. The correlation between the groups of the scale, in which the two half-test reliability was examined, was found to be statistically significant (*p*<.001) both in the factors and in the total scale (F1_r_: 0.703; F2_r_: 0.779; F3_r_: 0.739; F_Digital Dementia_: 0.895).

**Table 5:** Split-half test consistency and Cronbach α

Factor	Half	x	SD	r	Guttmann	Cronbach α
F1. Disruption in Daily Routine	1st Half	2.13	1.67	0.703	0.79	0.85
	2nd Half	3.02	1.66			
F2. Physical and Mental Decline	1st Half	3.08	1.80	0.779	0.84	0.87
	2nd Half	3.29	1.72			
F3. Emotional and Cognitive Decline	1st Half	2.47	1.78	0.739	0.77	0.87
	2nd Half	2.23	1.79			
Digital Dementia	1st Half	9.25	4.92	0.895	0.86	0.92
	2nd Half	9.76	5.10			

r : Spearman-Brown Coefficient

**Fig. 1:**
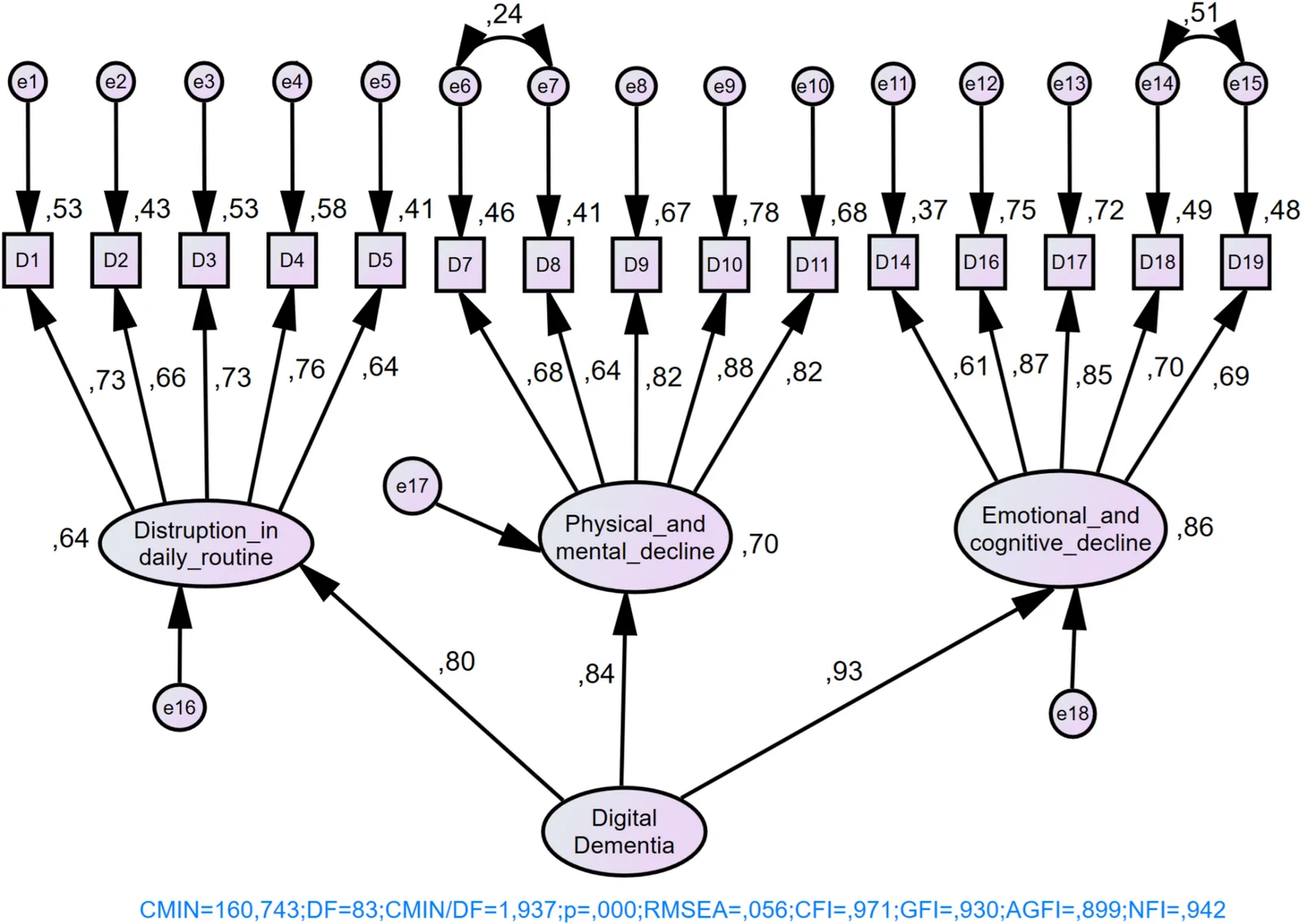
CFA findings regarding the 3-factor structure of the scale

When the paths associated with the 3-factor structure of the scale were evaluated, it was found that all sub-dimensions made a significant contribution to the scale (Fig. 1). Also, the items in the factors yielded a significant loading to the factor they were in. The factor loadings of the items in the Disruption in Daily Routine factor varied between 0.64 and 0.76, and between 0.64 and 0.88 in the Physical and Mental Impairment factor. The factor loadings of the items in the Emotional and Cognitive Impairment factor varied between 0.61 and 0.87. All items showed a significant relationship with the factors they were in (*p*< .001). To verify the structure of the scale, the item factor loadings as well as the fit indices of the model were evaluated along with whether they were at an acceptable level according to the literature data. Perfect and acceptable goodness of fit values in the literature and the goodness of fit values of the Digital Dementia Scale Adult Form are given in Table 6.

**Table 6:** The goodness of fit values of the scale and ranges accepted in the literature

Variable	χ2/df	RMSEA	CFI	GFI	AGFI	NFI
Dijital Demans	1.937	0.056	0.97	0.93	0.90	0.94
Good Fet	χ2 < 2	0 < RMSEA<0.05	0.95 ≤ CFI ≤ 1	0.95 ≤ GFI ≤ 1	AGFI>0.95	0.95 ≤ GFI ≤ 1
Acceptable Compatibility	χ2 < 5	0.05 < RMSEA<0.10	0.90 ≤ CFI≤0.95	0.90 ≤ CFI≤0.95	AGFI>0.90	0.90 ≤ CFI≤0.95

*RMSEA, *Root Mean Square Error of Approximation; *CFI, *Comparative Fit Indices; *GFI, *Goodness-of-Fit Index; *AGFI, *Adjusted Goodness of Fit Index; *NFI, *Normed Fit Index

The 3-factor structure of the Digital Dementia Scale Adult Form was tested with Second-Level Multi-Factor CFA. The items and dimensions in the sub-dimensions of the scale showed significant relationships with the structure they were in. When the Goodness of Fit values were also examined, it was found that it took the values x^2^ / df: 1.937, RMSEA: 0.056, CFI: 0.97, GFI: 0.93, AGFI: 0.90 and NFI: 0.94 and x^2^/df and CFI showed perfect Goodness of Fit values and RMSEA, GFI, AGFI, and NFI indices had acceptable Goodness of Fit values. Based on these results, it was concluded that the 3-factor structure of the scale was confirmed (Table 6).

When the Average Variance Extracted (AVE) and Composite Reliability (CR) values regarding the two-factor structure of the scale were examined, it was found that for the Disruption in Daily Routine factor, AVE = 0.50 and CR = 0.83, Physical and Mental Impairment factor AVE = 0.60 and CR = 0.88 and for the Emotional and Cognitive Impairment factor, AVE = 0.56 and CR = 0.86. The fact that the AVE values of both factors are above 0.50 indicates that the variance of the related items is explained at a significant level. Moreover, CR values above 0.70 demonstrate high internal consistency of the factors. In addition, the square root values of AVE being greater than the inter-factor correlation coefficients show that the factors can be distinguished from each other. These results provide strong evidence of construct validity for the measurement model of the scale (Table 7).

**Table 7:** Composite Reliability (CR) and Average Variance Extracted (AVE)

	AVE - CR						
		λ	λ^2^	1- λ^2^	n	AVE	CR
F1 Disruption in Daily Routine	I1	0.728	0.529984	0.470016	5	.50	.83
	I2	0.656	0.430336	0.569664			
	I3	0.73	0.5329	0.4671			
	I4	0.761	0.579121	0.420879			
	I5	0.64	0.4096	0.5904			
F2 Physical and Mental Decline	I7	0.679	0.461041	0.538959	5	.60	.88
	I8	0.641	0.410881	0.589119			
	I9	0.821	0.674041	0.325959			
	I10	0.885	0.783225	0.216775			
	I11	0.822	0.675684	0.324316			
F3 Emotional and Cognitive Decline	I14	0.611	0.373321	0.626679	5	.56	.86
	I16	0.865	0.748225	0.251775			
	I17	0.851	0.724201	0.275799			
	I18	0.7	0.49	0.51			
	I19	0.69	0.4761	0.5239			
	Discriminant Validity						
		AO	SD	F1	F2	F3	F4
	F1	1.30	.77	(.911)			
	F2	1.56	.83	.578**	(.938)		
	F3	1.19	.82	.608**	.685**	(.927)	

In the study, the relationships between screen time, age, and digital dementia were examined (Table 8). The analysis results indicated a positive and significant relationship between screen time and digital dementia (*r* = .632, *p* < .01). This finding suggests that as screen time increases, digital dementia symptoms also increase. In addition, a negative and significant relationship was found between age and digital dementia (*r* = − .357, *p* < .01). Accordingly, younger individuals experience digital dementia symptoms more intensely, while these symptoms decrease with increasing age. The negative relationship between screen time and age (*r* = − .273, *p* < .01) also revealed that younger individuals spend more time in front of screens.

**Table 8:** Digital dementia: the relationship between screen time and age

Variable	1	2	3
Screen Time (1)	1	− 0.273**	0.632**
Age (2)		1	− 0.357**
Digital Dementia (3)			1

**Correlation is significant at the 0.01 level (2-tailed)

## Discussion

The Digital Dementia Scale Adult Form was developed and validity and reliability analyses were conducted in the present study.

Content validity checks whether the measurement tool fully represents all the areas and contents it is supposed to cover, which is of critical importance, especially in social sciences, for the measurement tool to provide accurate and complete results [21]. Content validity is usually evaluated based on expert opinions. Experts in the field determine how much the items in the measurement tool cover the relevant concept and in which areas there are deficiencies [22]. According to the analysis, the Content Validity Index of the form as a whole was determined as 0.953. It was concluded that the form as a whole provided content validity because the obtained CVI value was greater than the CVR value of the form items (CVI > CVR).

Item total correlation is the correlation of the total scores of the scale/test with the scores of each item. If the item-total score correlation coefficient is < 0.30, it must be considered that there is a problem with the item and it must either be edited or removed from the scale [23, 24]. When the item total correlations of the items were evaluated, it was found that the reliability of all items was at a sufficient level in the study.

The Principal Components Analysis was used in the EFA conducted to determine the construct validity of the scale, and as the factor rotation technique, the Direct Oblimin Technique from the Oblique Rotation Techniques was used based on the assumption that the factors were associated with each other, and rotation was performed in 9 iterations. The results yielded a 3-factor structure, and the variance percentage was 65.7%, which is sufficiently large for a multi-factor scale. Another evidence for the construct validity of the scale was obtained by testing the obtained structure by using CFA, which revealed that the Goodness of Fit values of the 3-factor structure were at an acceptable level. Since Factor 1 reflects the deterioration in the individual’s daily life habits, it is called Disruption in Daily Routine, since Factor 2 includes statements about the individual’s physical and mental complaints, it is called Physical and Mental Decline, since Factor 3 includes statements about affective and cognitive deterioration, it is called Emotional and Cognitive Decline. It was found that all items in the scale showed a significant relationship with the factors they were included in.

A lower group and upper group comparison was used for the internal validity of the 3-factor structure of the scale, and it was found that the scale accurately distinguished individuals with high and low levels of digital dementia, in other words, it had internal validity.

Reliability can be defined as the accuracy with which test or scale results uncover the phenomenon associated with the conceptual structure and the similar results when the measurement tool is applied in different places, at different times, and on different populations selected from the same main population [24]. Internal consistency must first be ensured in Likert-type scales. Internal consistency is associated with the degree to which the items that constitute the scale are compatible with each other. In the development of measurement tools that will be used to measure cognitive and affective characteristics, reliability coefficients can be calculated by using different methods. One of these methods is Cronbach’s Alpha Reliability. Although it is desired for the reliability coefficient to be considered sufficient in a Likert-type scale to be above 0.70, it must be as close to “1” as possible [25, 26]. For scales, it is recommended that Cronbach’s α Value below 0.60 is unacceptable, between 0.60 and 0.65 is not desirable, between 0.65 and 0.70 is minimally acceptable, between 0.70 and 0.80 is significant, between 0.80 and 0.90 is very good, and if it is much above 0.90, the researcher must consider shortening the scale [25]. In the present study, the reliability of the scale was evaluated by calculating Cronbach’s α Reliability Coefficient and Split-Half Test Consistency. The Cronbach’s α Reliability Coefficient was calculated as 0.92, and it was concluded that the reliability of the scale was at a sufficient level. According to the literature data, a Cronbach’s α value of 0.60 and below is “unacceptable”, 0.60–0.65 is “undesirable”, 0.65–0.70 is “least acceptable”, 0.70–0.80 is “significant”, 0.80–0.90 is “very good”, and well above 0.90 is “the scale must be shortened” [25].

The positive and significant relationship between screen time and digital dementia indicates that excessive screen use leads to impairments in cognitive, emotional, and physical functions. It can be suggested that individuals who spend long periods in front of screens experience digital dementia symptoms such as attention deficits, memory problems, and mental and physical fatigue more intensely. The negative relationship between age and digital dementia demonstrates that younger individuals spend more time with digital technologies and are therefore in the risk group. Indeed, the higher screen time among younger people results in more pronounced digital dementia symptoms in this age group. These findings show that screen time not only varies inversely with age but also constitutes one of the most important predictors of digital dementia.

### Limitations

The following limitations should be considered when interpreting the results. First, the data were collected using self-report instruments. These are susceptible to subjective biases such as lack of introspection. Second, our sample consisted only of adults, so the results cannot be transferred to adolescents without further studies. Conversely, this metadological study, characterised by a cross-sectional design, was constrained by the specific time. It is recommended that the assessment of digital dementia in adults be further developed in parallel with rapidly changing technological developments, such as artificial intelligence. Finally, this study provides only initial validity and reliability evidence for the DDAF; the scale requires additional psychometric validation in longitudinal use, different cultural settings, and larger samples.

## Conclusion

The validity and reliability of the Digital Dementia Scale Adult Form for adults (18 years and older) in Turkish culture were performed in this study. As a result of the content validity, structural validity, and reliability analyses, it was found that the Digital Dementia Scale Adult Form had sufficient reliability and validity and could make effective measurements in Turkish culture (Appendix 1, Appendix 2, Appendix 3).

## Appendix 1

**Scale Guideline**

The Digital Dementia Scale Adult Form is a measurement tool consisting of 3 factors and 15 items in a 5-point Likert style (Never: “0”, Rarely: “1”, Sometimes: “2”, Frequently: “3” and Always: “4”). There are no reverse-scored items in the scale. The total variance explained by the scale is 65.7% and the Cronbach α Reliability Coefficient calculated for the entire scale is .92. The scale is scored by summing the scores of the sub-dimensions and a score between 0-60 is obtained, which must be standardized to 0-100 points according to the standardization rules specified in the guidelines (if there is a digit after the decimal point, it must be rounded to the nearest integer) and analyses must be conducted on the standardized scores in future studies.

**Factor 1 (Disruption in Daily Routine):** This factor includes disruptions in the individual’s daily routines because of excessive use of digital devices and prolonged screen time and consists of items 1-5. The variance explained by this factor is 48.1% and the Cronbach α reliability coefficient of the factor is .85. The score that can be obtained from this factor varies between 0-20.

**Factor 2 (Physical and Mental Decline):** This factor shows the decline in the physical and mental health of the individual because of excessive use of digital tools and prolonged screen time and consists of 6-10 items. The variance explained by this factor is 9.6% and the Cronbach α reliability coefficient of the factor is .87. The score that can be obtained from this factor varies between 0-20.

**Factor 3 (Emotional and Cognitive Decline):** This factor shows the decline in the emotional and cognitive health of the individual because of excessive use of digital tools and prolonged screen time and consists of items 11-15. The variance explained by this factor is 7.3% and the Cronbach α reliability coefficient of the factor is .87. The score that can be obtained from this factor varies between 0-20.

**Standardization:** Standardization was made in the scoring of this scale to make comparisons between samples easily and consistently in studies using the scale and in adaptation studies to be conducted.

The following formula must be used to standardize the scores obtained from the scale to 100.

$$\mathrm{Standardized}\;\mathrm{Score}\;=\;\left(\frac{\mathrm{Original}\;\mathrm{Score}-\mathrm{Minimum}\;\mathrm{score}\;\mathrm{of}\;\mathrm{scale}\;\left(0\right)}{\mathrm{Range}\;\mathrm{of}\;\mathrm{scale}\;\left(60\right)}\right)\times100$$

This formula is used to express any original score (e.g., the score obtained on the scale) in a range of “0” to “100”.

For example, if a person scores 30 on the scale, his standardized score can be calculated as follows.

$$\mathrm{Standardized}\;\mathrm{Score}\;=\;\left(\frac{30\;-\;0}{60}\right)\times100=50$$

In this case, the standardized score of someone who scores 30 on the scale is 50. The standardized form of the scores obtained from the scale must be used in future studies.

## Appendix 2

**Table:**

	DIGITAL DEMENTIA SCALE ADULT FORM Digital Device: Digital devices such as smartphones, computers, tablets, television, etc. Various situations that occur depending on “Spending time in front of a screen or using a digital device” are listed below. Please mark how often you experience these situations in a way that best reflects you.	Never (0)	Rarely (1)	Sometimes (2)	Frequently (3)	Always (4)
1	Postponing daily tasks.					
2	Staying in front of the screen for long periods.					
3	Irregular, excessive, or inadequate eating and drinking.					
4	Irregular, excessive, or inadequate sleep.					
5	Spending time in front of the screen until late hours.					
6	Waist, back, or neck pain.					
7	Headaches or eyestrain.					
8	Difficulty concentrating.					
9	Mental fatigue.					
10	Physical fatigue.					
11	Decreased face-to-face social interaction.					
12	Feeling more restless or anxious.					
13	Feeling more depressed.					
14	Difficulty remembering everyday information or tasks.					
15	Difficulty learning or remembering new information.					

## Appendix 3

**Table:**

	DİJİTAL DEMANS ÖLÇEĞİ: YETİŞKİN FORMU Dijital Cihaz: Akıllı telefon, Bilgisayar, Tablet, Televizyon vb. dijital araçlar Aşağıda "Ekran başında kalmanıza ya da dijital cihaz kullanımınıza" bağlı olarak ortaya çıkan çeşitli durumlar sıralanmıştır. Bu durumları ne sıklıkla yaşadığınızı sizi en iyi yansıtacak şekilde işaretleyiniz.	Hiçbir Zaman (0)	Nadiren (1)	Bazen (2)	Sık Sık (3)	Her Zaman (4)
1	Günlük işleri erteleme.					
2	Uzun süre ekran karşısında kalma.					
3	Düzensiz, aşırı ya da yetersiz yeme içme.					
4	Düzensiz, aşırı ya da yetersiz uyku.					
5	Geç saatlere kadar ekran başında vakit geçirme.					
6	Bel, sırt ya da boyun ağrısı.					
7	Baş ağrısı ya da göz yorgunluğu.					
8	Dikkati toplamada güçlük.					
9	Zihinsel yorgunluk.					
10	Fiziksel yorgunluk.					
11	Yüz yüze sosyal etkileşimin azalması.					
12	Kendini daha huzursuz ya da kaygılı hissetme.					
13	Kendini daha depresif hissetme.					
14	Gündelik bilgileri ya da görevleri hatırlamada güçlük.					
15	Yeni bilgileri öğrenme ya da hatırlamada güçlük.					
